# Clinical relevance of circulating mucosal-associated invariant T cell levels and their anti-cancer activity in patients with mucosal-associated cancer

**DOI:** 10.18632/oncotarget.11187

**Published:** 2016-08-10

**Authors:** Eun Jeong Won, Jae Kyun Ju, Young-Nan Cho, Hye-Mi Jin, Ki-Jeong Park, Tae-Jong Kim, Yong-Soo Kwon, Hae Jin Kee, Jung-Chul Kim, Seung-Jung Kee, Yong-Wook Park

**Affiliations:** ^1^ Department of Laboratory Medicine, Chonnam National University Medical School and Hospital, Gwangju, Republic of Korea; ^2^ Department of Surgery, Chonnam National University Medical School and Hospital, Gwangju, Republic of Korea; ^3^ Department of Rheumatology, Chonnam National University Medical School and Hospital, Gwangju, Republic of Korea; ^4^ Department of Pulmonary and Critical Care Medicine, Chonnam National University Medical School and Hospital, Gwangju, Republic of Korea; ^5^ Heart Research Center, Chonnam National University Hospital, Gwangju, Republic of Korea

**Keywords:** cytotoxicity, chemokine, migration, mucosal-associated invariant T cells, mucosal-associated cancer

## Abstract

Mucosal-associated invariant T (MAIT) cells are an antimicrobial MR1-restricted T cell subset and play an important role in immune defense response to bacteria. However, little is known about the role of MAIT cells in cancer. The aims of this study were to examine the level and function of MAIT cells in cancer patients and to evaluate the clinical relevance of MAIT cell levels. Ninety-nine patients with cancer and 20 healthy controls were included in this study. Circulating MAIT cell levels were significantly reduced in patients with mucosal-associated cancers (MACs), such as gastric, colon and lung cancers, but their capacities for IFN-γ, IL-17, or TNF-α production were preserved. This MAIT cell deficiency was significantly correlated with N staging and carcinoembryonic antigen level. Percentages of MAIT cells were significantly higher in cancer tissue than in peripheral blood and immunofluorescent labeling showed MAIT cell infiltration into colon cancer tissues. Circulating MAIT cells exhibited high levels of CCR6 and CXCR6, and their corresponding chemokines, such as CCL20 and CXCL16, were strongly expressed in colon cancer tissues. Activated MAIT cells not only had lymphokine-activated killer activity, but they also had direct cytotoxicity on K562 cells via degranulation of granzyme B and perforin. This study primarily demonstrates that circulating MAIT cells are reduced in MAC patients due to migration to mucosal cancer tissues and they have the potential to kill cancer cells. In addition, this circulating MAIT cell deficiency is related to the degree of cancer progression in mucosal tissues.

## INTRODUCTION

Chronic inflammatory stimuli that induce infiltration of immune cells into tissue sites have been known to be one of the key risk factors for initiating and developing cancer [1]. The development in oncoimmunology was based on the identification of highly diverse tumor-infiltrating lymphocyte (TIL) populations, such as natural killer (NK) and CD8^+^ cells, that have antagonistic function in the tumor niche [2]. TIL subsets have been shown to be capable of recognizing and targeting transformed cells, thus leading to their elimination, and these properties have also been validated in murine models [2]. The TIL subsets are directly cytotoxic to cancer cells via intracellular cytotoxic granules, containing perforin and granzymes, and are also able to indirectly initiate anti-tumor immune responses by secreting various proinflammatory cytokines such as interferon (IFN)-γ, tumor necrosis factor (TNF)-α, and interleukin (IL)-17 [3, 4]. Moreover, invariant natural killer T (iNKT) cells, one of the two invariant T cell subsets, are unique T cell subpopulation capable of regulating tumor immunity, transplantation immunity, allergy, autoimmunity and microbial immunity [5, 6]. Especially, the capacity of iNKT cells to modify the immune microenvironment influences the ability of the host to control tumor growth, and indeed, the identification of strong iNKT-cell agonists, such as α-galactosylceramide (α-GalCer) and its analogues, has led to the development of synthetic lipids that have shown potential in vaccination and treatment against cancers [7]. In the initial anti-tumor response after stimulation with α-GalCer and dendritic cells (DCs), iNKT cells immediately produce large amounts of IFN-γ, which acts on DCs, NK cells, and neutrophils in the innate immune system to eliminate major histocompatibility complex (MHC) negative tumor target cells and, at the same time, also on CD8 cytotoxic T cells and CD4 Th1 cells to kill MHC positive tumor cells, resulting in tumor eradication [8].

Mucosal-associated invariant T (MAIT) cells, another invariant T cell subset, are an evolutionarily conserved antimicrobial MR1-restricted T cell subset, defined as CD3^+^TCRδγ^−^ Vα7.2^+^CD161^high^ or CD3^+^TCRδγ^−^ Vα7.2^+^ IL-18Rα^+^ cells [9, 10]. MAIT cells play an important role in immune defense response to bacteria and yeast via rapidly producing Th1/Th17 cytokines, such as IFN-γ and IL-17 [9, 10]. Similarity of MAIT cells to iNKT cells makes us to be curious about their ability as effectors of tumor-infiltrating lymphocyte subset. A prime example of one such hypothesis that MAIT cells augment anti-tumor responses is based on their characteristics producing IFN-γ, which acts on NK cells to eliminate MHC negative tumors and also on CD8 cytotoxic T cells to kill MHC positive tumors. Since human MAITs show the higher level in circulation than iNKT cell population [11, 12], they may have enough potential as a useful tool in immune-cell therapy. Quiet recently, a few reports announced that MAIT cells were infiltrated in tumors of kidney, brain, and colon [13, 14]. Mainly, MAIT cells have been known to be associated with infectious diseases [15–21] and autoimmune disorders [22–24]. However, the role of MAIT cells in cancer has not been elucidated yet.

MAIT cells are abundant in peripheral blood, mucosal tissues in the gastrointestinal tract and lung, mesenteric lymph nodes, and liver [9, 10]. This preferential tissue tropism of MAIT cells can be explained by the specific chemokine receptor expression such as CCR6, CCR9 and, CXCR6 [9]. Moreover, CCL20, the ligand for CCR6, has been known to be highly expressed in gastric cancer, colorectal cancer, and lung cancer [25–27]. These types of cancers, which are known to mainly involve the mucosal tissue and shown CCL20 expression, were designated as mucosal-associated cancers (MACs) in the present study, while the other cancers, such as thyroid, breast, and liver cancer, were designated as non-MACs. Accordingly, the aims of this study were to examine the level and function of MAIT cells in a variety of cancer types including MACs and non-MACs, to evaluate the clinical relevance of MAIT cell levels, and finally to determine the immune defense mechanism of MAIT cells against cancers.

## RESULTS

### Reduced numbers of circulating MAIT cells in MAC patients

**Figure 1:**
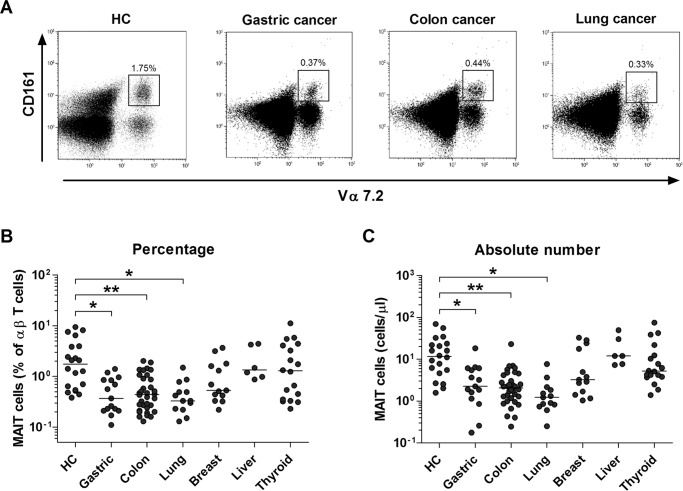
Decreased circulating MAIT cell numbers in the peripheral blood of MAC patients Freshly isolated PBMCs from 20 HCs, 15 patients with gastric cancer, 34 patients with colon cancer, 13 patients with lung cancer, 13 patients with breast cancer, 6 patients with liver cancer and 18 patients with thyroid cancer were stained with APC-Alexa Fluor 750-conjugated anti-CD3, FITC-conjugated anti-TCR γδ, APC-conjugated anti-TCR Vα7.2 and PE-Cy5-conjugated anti-CD161 mAbs and then analyzed by flow cytometry. Percentages of MAIT cells were calculated within a αß T cell gate. **A.** Representative MAIT cell percentages as determined by flow cytometry. **B.** MAIT cell percentages among peripheral blood αß T cells. **C.** Absolute MAIT cell numbers (per microliter of blood). Symbols (•) represent individual subjects; horizontal bars show the median. *, *P* < 0.05, **, *P* < 0.005 by ANCOVA test. MAC, Mucosal-associated cancer.

The percentages and absolute numbers of MAIT cells in the peripheral blood samples from 15 gastric cancer patients, 34 colon cancer patients, 13 lung cancer patients, 13 breast cancer patients, 6 liver cancer patients, 18 thyroid cancer patients, and 20 HCs were determined by flow cytometry (Table 1). All comparisons of percentages and absolute numbers of MAIT cells were performed by analysis of covariance after adjusting for age and sex using Bonferroni correction for multiple comparisons, as described in the ‘Methods’ section. MAIT cells were defined as CD3+γδ- T cells expressing TCR Vα7.2 and CD161^high^ (Figure 1A). Percentages of MAIT cells were significantly lower in gastric, colon and lung cancer patients than in healthy controls (HCs) (median: 0.37% versus 1.75% [*P* < 0.05]; 0.44% versus 1.75% [P < 0.005]; and 0.33% versus 1.75% [*P* < 0.05], respectively; Figure 1B). Gastric, colon and lung cancer patients had significantly lower absolute numbers of MAIT cells as compared with HCs (median: 2.25 cells/μl versus 11.6 cells/μl [*P* < 0.05]; 2.06 cells/μl versus 11.6 cells/μl [*P* < 0.005]; and 1.23 cells/μl versus 11.6 cells/μl [*P* < 0.05], respectively, Figure 1C). However, no significant differences were observed in the percentages and absolute numbers of MAIT cells between breast, liver, or thyroid cancer patients and HCs. This study categorized cancer types based on the involvement in mucosal tissue; gastric, colon, and lung cancers were categorized into MACs; thyroid, breast, and liver cancers were categorized into non-MACs. Circulating MAIT cell levels were compared between the two cancer groups, thus showing a significant decline in MAIT cell levels in MACs compared to non-MACs (median 0.41% versus 1.20% [*P* < 0.05]; data not shown).

**Table 1:** Clinical and laboratory characteristics of 99 patients with cancer

male/female, n	52/47
Age (year), mean ± SD	60.9 ± 14.3
Cancer group, n	
Stomach cancer	15
Colon cancer	34
Lung cancer	13
Breast cancer	13
Liver cancer	6
Thyroid cancer	18
Tumor diameter (maximum, mm), mean ± SD	26.6 ± 23.5
T Staging*, n (%)	
T0	4 (4.0)
T1	33 (33.3)
T2	15 (15.2)
T3	22 (22.2)
T4	7 (7.1)
N Staging*, n (%)	
N0	76 (76.8)
N1	16 (16.2)
N2	7 (7.1)

Abbreviations: n, number; SD = standard deviation.

* The staging of T and N was following the TNM Staging System defined by the American Joint Committee on Cancer and the Union for International Cancer Control. The T staging is based on the extent of the tumor and the N staging is based on the extent of spread to the lymph nodes. All cases included in this study were M0 staging.

**Figure 2:**
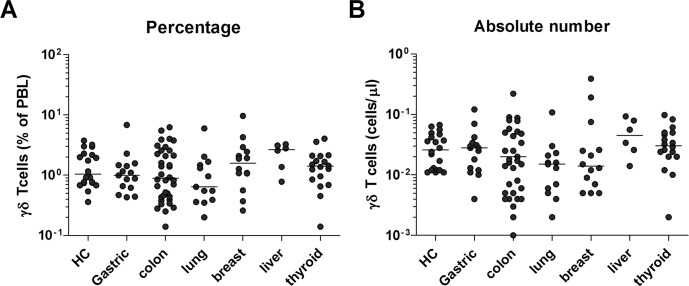
Frequencies of γδ T cells in the peripheral blood of cancer patients Freshly isolated PBMCs from 20 HCs, 15 patients with gastric cancer, 34 patients with colon cancer, 13 patients with lung cancer, 13 patients with breast cancer, 6 patients with liver cancer and 18 patients with thyroid cancer were stained with APC-Alexa Fluor 750-conjugated anti-CD3 and FITC-conjugated anti-TCR γδ mAbs and then analyzed by flow cytometry. **A.** Percentages of γδ T cells. **B.** Absolute γδ T cell numbers (per microliter of blood). Symbols (•) represent individual subjects; horizontal bars show the median.

To determine whether the decline in MAIT cell levels is due to true decrease in numbers or dilution effect by infection-reactive mainstream T cells, we next investigated frequencies of γδ T cells by flow cytometry. The percentages and absolute numbers of γδ T cells in peripheral blood were found to be similar between the cancer patients and HCs, suggesting that the decline in cell levels is specific to MAIT cells (Figure 2).

### Relationship between circulating MAIT cell levels and clinical parameters in MAC patients

To evaluate the clinical relevance of MAIT cell levels in MAC patients, we investigated the correlation between MAIT cell numbers in peripheral blood and clinical parameters using Spearman's correlation analysis (Table 2). The analysis showed that absolute MAIT cell numbers were significantly correlated with N staging, lymphocyte count, neutrophil count, hemoglobin, and carcinoembryonic antigen (CEA) levels (all, *P* < 0.05). In addition, tumor size tended to correlate with MAIT cell numbers, which did not reach statistical significance, probably due to the small sample size (Supplementary Table 1). However, no significant correlations were observed between MAIT cell numbers and age, T staging, leukocyte count, monocyte count, platelet count, AST, ALT, BUN, creatinine, total protein, albumin, or CRP levels. These results suggest that circulating MAIT cell deficiency may reflect the degree of cancer progression in mucosal tissues from MAC patients.

**Table 2:** Spearman's correlation coefficients for MAIT cell numbers with respect to clinical and laboratory findings in MAC patients

Variable	Correlation coefficient (γs)	*P* value
Age	−0.033	0.799
T staging	−0.160	0.252
N staging	−0.302	0.030
Tumor size	−0.251	0.070
Leukocyte count (cells/μL)	−0.152	0.238
Lymphocyte count (cells/μL)	0.346	0.006
Monocyte count (cells/μL)	−0.175	0.173
Neutrophil count (cells/μL)	−0.270	0.034
Hemoglobin (g/dL)	0.344	0.006
Platelet count (103 cells/μL)	−0.044	0.732
AST (U/L)	0.027	0.833
ALT (U/L)	0.006	0.961
BUN (mg/dL)	−0.189	0.141
Creatinine (mg/dL)	−0.070	0.587
Total protein (g/dL)	−0.051	0.718
Albumin (g/dL)	0.155	0.262
CRP (mg/dL)	−0.241	0.102
CEA (ng/mL)	−0.410	0.008

Abbreviations: AST, Aspartate transaminase; ALT, Alanine transaminase; BUN, Blood urea nitrogen; CRP, C-reactive protein; CEA, Carcinoembryonic antigen; MAC, Mucosal-associated cancer.

### Preserved production of proinflammatory cytokines in MAIT cells of MAC patients

**Figure 3:**
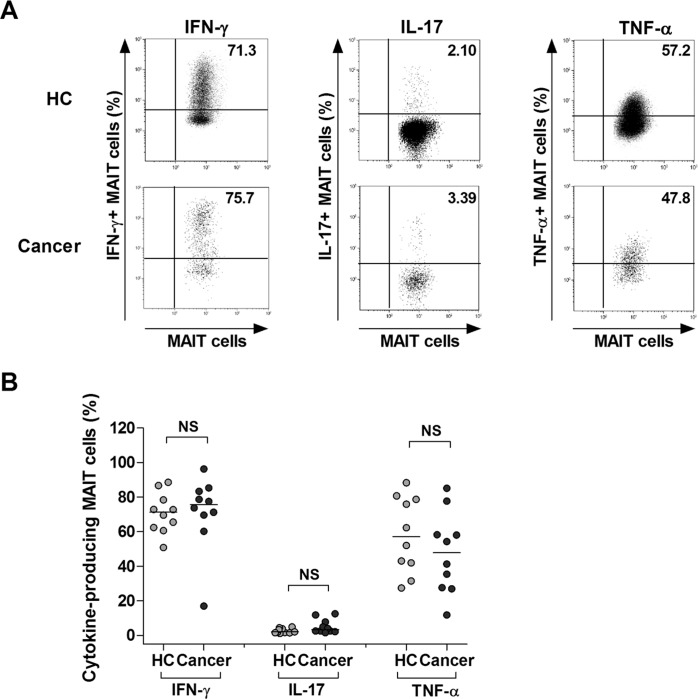
Expression of IFN-γ, IL-17 and TNF-α in MAIT cells of MAC patients Freshly isolated PBMCs (1 × 10^6^/well) were incubated for 4 hours in the presence of PMA (100 ng/ml) and IM (1 μM). **A.** Representative IFN- γ, IL-17 and TNF-α expression in the MAIT cell population were determined by intracellular flow cytometry after stimulation with PMA and IM. Data in **B.** were obtained from 10 HCs and 10 MAC patients, including 4 patients with gastric cancer, 3 patients with colon cancer and 3 patients with lung cancer. Symbols represent individual subjects; horizontal bars indicated the median. MAC, Mucosal-associated cancer; NS, not significant.

Proinflammatory cytokines, including IFN-γ, IL-17, and TNF-α, are known to have anti-neoplastic and anti-microbial activities [28–33]. To investigate expression of these cytokines in MAIT cells, we incubated PBMCs from 4 gastric cancer patients, 3 colon cancer patients, 3 lung cancer patients, and 10 HCs for 4 hours in the presence of PMA and IM; then the expression of IFN-γ, IL-17, and TNF-α in the MAIT cell population was examined at the single-cell level by intracellular flow cytometry (Figure 3A). IFN-γ+, IL-17+, or TNF-α+ MAIT cell levels were comparable between the patients and HCs (Figure 3B). Collectively, these results indicate that circulating MAIT cells seem to preserve anti-cancer cytokines even in MAC patients.

### Migration of MAIT cells into colon cancer tissues

**Figure 4:**
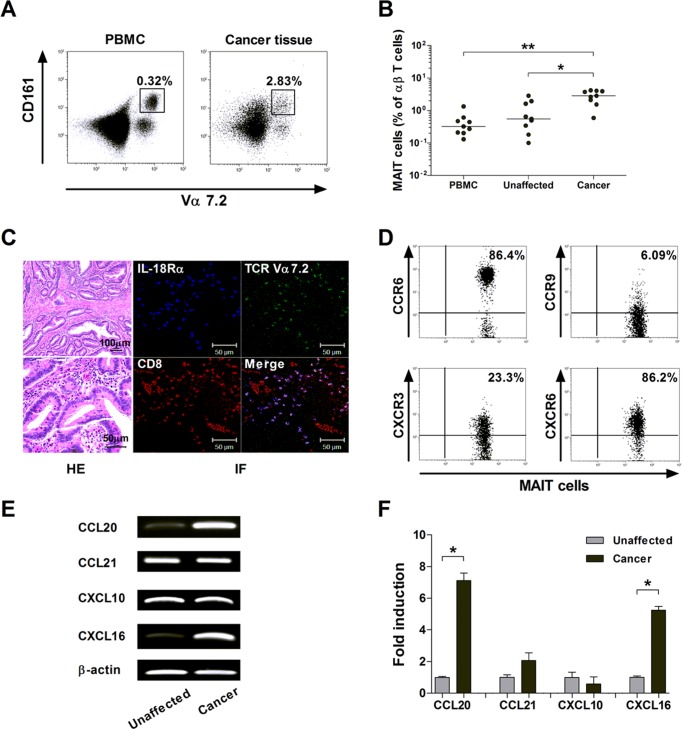
Migration of MAIT cells into colon cancer tissues **A.** and **B.** Increased MAIT cell levels in colon cancer tissues. A representative paired sample was obtained from a colon cancer patient and MAIT cell percentages were determined by flow cytometry **A.** Data in **B.** were obtained from 9 patients with colon cancer. Symbols represent individual patients. *, *P* < 0.05, **, *P* < 0.005 by Wilcoxon matched-pairs signed rank test. **C.** Infiltration of MAIT cells into colon cancer tissues. Tissue sections from patients with colon cancer were processed for hematoxylin-eosin (HE) and immunofluorescent (IF) staining using anti-CD8 (red), anti-TCR Vα7.2 (green), anti-IL-18Rα (blue) antibodies. Scale bars in C = 50 and 100 μm. **D.** Expression of tissue-homing chemokine receptors in circulating MAIT cells. Representative staining for chemokine receptors on peripheral blood MAIT cells was obtained from a colon cancer patient and then analyzed by flow cytometry. **E.** and F. Expression of mRNA for chemokines in the colon cancer and unaffected colon tissues. Reverse transcription-polymerase chain reaction (RT-PCR) **E.** and real-time PCR **F.** were performed to determine the mRNA expression of each gene. Data in **F.** were obtained from 3 patients with colon cancer. Values are expressed as the mean ± SEM. *, *P* < 0.01 by paired t test.

To examine whether circulating MAIT cell deficiency in MAC patients might be associated with accumulation of MAIT cells in cancer tissues, we obtained paired samples of peripheral blood and cancer tissue from 9 colon cancer patients, and MAIT cell levels were determined by flow cytometry (Figure 4A). Percentages of MAIT cells were significantly higher in cancer tissue than in peripheral blood and unaffected tissue (median: 2.83% versus 0.32%, *P* < 0.005; 2.83% versus 0.55%, *P* < 0.05, respectively; Figure 4B). Next, the tissue sections were stained by immunofluorescent labeling using anti-CD8, anti-TCR Vα7.2 and anti-IL-18Rα, and then examined by confocal microscopy. Cells that stained positively for CD8, TCR Vα7.2 and IL-18Rα were present in the cancer tissues, which suggest that MAIT cells can infiltrate into colon cancer (Figure 4C).

To determine whether MAIT cells have the capability of trafficking into peripheral target tissue, the pattern of tissue-homing chemokine receptor expression in circulating MAIT cells was determined by flow cytometry. MAIT cells in peripheral blood exhibited high levels of CCR6 and CXCR6, intermediate levels of CXCR3, and low levels of CCR9 (Figure 4D). We also analyzed the pattern of each corresponding chemokine expression in paired samples of colon cancer and its adjacent unaffected tissues by RT-PCR and real-time PCR. Notably, CCL20 and CXCL16 mRNA were found to be more strongly expressed in cancer tissue than in unaffected tissue. CCL21 and CXCL10 transcript levels were comparable between the cancer and unaffected tissues (Figure 4E and 4F).

**Figure 5:**
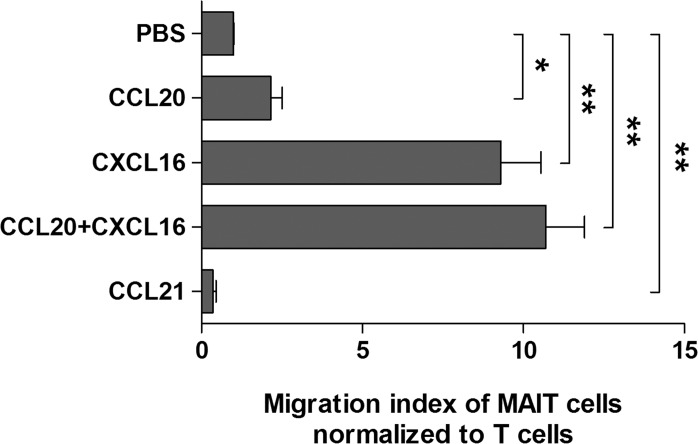
Migration of MAIT cells induced by chemokines Purified T cells (5 × 10^5^ cells/well) were added to upper chambers of Transwell plates. rhCCL20 (200 ng/ml), rhCXCL16 (200 ng/ml), mixture of rhCCL20 and rhCXCL16 (200 ng/ml), rhCCL21 (200 ng/ml), and PBS as a negative control were added to lower chambers. Plates were incubated for 90 minutes at 37°C. Migrated cells were stained with APC-Alexa Fluor 750-conjugated anti-CD3, FITC-conjugated anti-TCR γδ, APC-conjugated anti-TCR Vα7.2 and PE-Cy5-conjugated anti-CD161 mAbs and then counted using Neubauer chamber and flow cytometer. Migration index was calculated by the number of cells that migrated in the presence of each chemokine divided by the number of cells that migrated in the presence of PBS. The migration index of MAIT cells was normalized to the migration index of T cells. Values are expressed as the mean ± SEM. Data were obtained from 5 HCs. *, *P* < 0.05, **, *P* < 0.005 by paired t test.

To determine the direct effect of chemokines on MAIT cell migration, a Transwell migration assay was performed. Migration of MAIT cells was significantly increased in the presence of CCL20, CXCL16, or mixture of CCL20 and CXCL16 as compared with phosphate buffered saline (PBS) as a negative control (migration index of MAIT cells normalized to T cells, mean ± SEM: 2.16 ± 0.35 versus 0.99 ± 0.02 [*P* < 0.05]; 9.30 ± 1.25 versus 0.99 ± 0.02 [*P* < 0.005], and 10.7 ± 1.20 versus 0.99 ± 0.02 [*P* < 0.005], respectively), but not in the presence of CCL21 (0.35 ± 0.10 versus 0.99 ± 0.02, *P* < 0.005; Figure 5). Taken together, these data clearly indicate that MAIT cells are circulating lymphocytes with tissue tropism and can migrate from peripheral blood into cancer tissue via this chemokine-chemokine receptor axis.

### Cytotoxicity of activated MAIT cells

**Figure 6:**
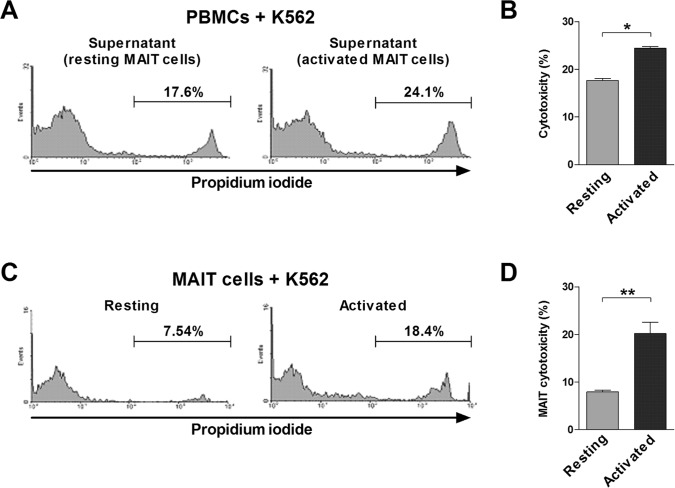
Cytotoxicity of MAIT cells **A.** and **B.** LAK activity of MAIT cells. Freshly isolated PBMCs (1 × 10^6^/well) were cocultured with K562 cells for 4 hours in the presence of the supernatant harvested from purified MAIT cells stimulated for 12 hours with or without PMA (100 ng/ml) and IM (1 μM). **C.** and **D.** Cytotoxicity of purified MAIT cells. Following stimulation with or without PMA (100 ng/ml) and IM (1 μM), MAIT cells (1 × 10^6^/well) were then cocultured with K562 cells. LAK activity **A.** and cytotoxicity **C.** were then determined as the percentage of apoptotic K562 cells and respresentative flow cytometry results are presented. Data in **B.** and **D.** were obtained from 5 HCs. Values are expressed as the mean ± SEM. *, *P* < 0.05, **, *P* < 0.005 by paired t test.

To examine whether MAIT cells have potential anti-cancer activity, we used K562 cell lysis assay as an experimental model. To determine lymphokine-activated killer (LAK) activity of MAIT cells, purified MAIT cells were incubated in the presence or absence of PMA and IM, and then culture supernatants were harvested as described in the ‘Methods’ section. LAK activities of PBMCs were evaluated by flow cytometry and determined at an E:T cell ratio of 20:1. The LAK activities were significantly higher in the supernatant of activated MAIT cells than in that of resting MAIT cells (mean ± SEM: 24.4 ± 0.37% versus 17.7 ± 0.45%, *P* < 0.05; Figure 6A and 6B). To determine whether MAIT cells have direct cytotoxicity on K562 cells, purified MAIT cells were cocultured with K562 cells following stimulation with or without PMA and IM. MAIT cytotoxicities were then evaluated by flow cytometry at an E:T ratio of 40:1. The cytotoxicities were significantly higher in activated MAIT cells as compared with resting MAIT cells (mean ± SEM: 20.2 ± 2.38% versus 7.95 ± 0.35%, *P* < 0.005; Figure 6C and 6D). Collectively, these findings suggest that activated MAIT cells have LAK activity and direct cytotoxicity on cancer cells.

### Cytotoxic profile expression by MAIT cells

**Figure 7:**
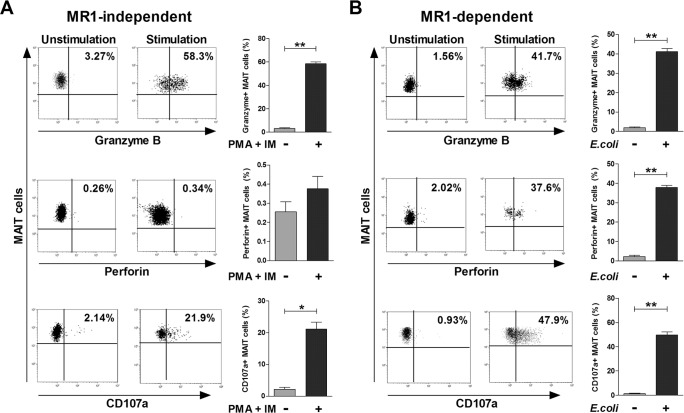
Cytotoxic profile of MAIT cells **A.** MR1-independent activation. Freshly isolated PBMCs (1 × 10^6^/well) were incubated for 12 hours in the presence or absence of PMA (100 ng/ml) and IM (1 μM). **B.** MR1-dependent activation. Freshly isolated PBMCs (1 × 10^6^/well) were cocultured for 24 hours in the presence or absence of *E. coli*-infected THP-1 cells. Granzyme B, perforin and CD107a expressions in MAIT cells were determined by flow cytometry as described in the ‘Patients and Methods’ section. Data in (A and B) were obtained from 5 HCs. Values are expressed as the mean ± SEM. *, *P* < 0.001, **, *P* < 0.0001 by paired t test.

To confirm the direct cytotoxic effect of MAIT cells against K562 cells, we investigated the expression of cytotoxic granules (i.e., granzyme B and perforin) and degranulation marker (CD107a). Upon stimulation with PMA and IM in an MR1-independent manner, granzyme B and CD107a expression levels in MAIT cells were found to be upregulated as compared with the unstimulated status (mean ± SEM: 58.5 ± 1.58% versus 3.19 ± 0.63% [*P* < 0.0001] and 21.1 ± 2.23% versus 2.20 ± 0.64% [*P* < 0.001], respectively; Figure 7A). However, no significant changes in perforin expression levels were observed. After stimulation with *E. coli*-infected THP-1 cells in an MR1-dependent manner, granzyme B, perforin and CD107a expressions in MAIT cells were increased more significantly as compared with the unstimulated status (mean ± SEM: 41.1 ± 1.56% versus 1.92 ± 0.35% [*P* < 0.0001], 37.9 ± 1.08% versus 2.30 ± 0.56% [*P* < 0.0001], and 49.8 ± 2.54% versus 1.33 ± 0.37% [*P* < 0.001], respectively; Figure 7B). Taken together, our data suggest that MAIT cytotoxicity may be mediated via degranulation of cytotoxic granules in both MR1-independent and MR1-dependent manners.

## DISCUSSION

The present study is the first attempt to reveal the relationship between MAIT cells and various types of solid tumors focused on highly prevalent cancers. We showed that circulating MAIT cells, capable of producing anti-cancer cytokines, are reduced particularly in MAC patients and their deficiencies are correlated with prognostic parameters such as N staging and CEA. This study shows that MAIT cells have the potential to migrate to mucosal tissues in the gut and they are accumulated in the region of colon cancer tissues. We also demonstrated that activated MAIT cells not only have LAK activity on cancer cells but they also have direct cytotoxicity, which may be mediated via degranulation of cytotoxic granules in both MR1-independent and MR1-dependent manners. Here, we suggest a new role of MAIT cells as an immune barrier to MACs.

Until now, MAIT cells have been found to be present in renal, brain, and colorectal tumors [13, 14]. A previous study demonstrated that circulating MAIT cells were not different between patients with colorectal cancer and healthy controls [14]. In contrast, our data showed that circulating MAIT cells were markedly declined in patients with colorectal cancer than in healthy controls, which was consistent with a recent report [34]. Furthermore, we found that circulating MAIT cells were also reduced in patients with gastric cancer and patients with lung cancer. All of these cancers were associated with the mucosal barrier and were accordingly categorized as MACs in the present study. On the contrary, circulating MAIT cells were not declined in patients with breast, thyroid or liver cancers, which were classified as non-MACs. These findings confirmed the observations of a previous study showing that circulating MAIT cell levels were comparable between healthy donors and patients with breast cancers [10]. This led us to speculate that MAIT cells might be redistributed to the mucosal tissues, especially in MACs, which results in deficiency of circulating MAIT cells. The discrepancy between the previous study and our study might be due to cohort selection bias including age, sex, and race. Our previous study showed that downward shift in MAIT cell frequency and Th1 to Th2 shift in cytokine profile were correlated with aging [11]. In the present study, comparative analysis of MAIT cell frequency between cancer patients and controls was compensated by age and sex. A further study targeting large scale populations needs to be performed to validate changes in MAIT cell levels in the peripheral blood of patients with various types of cancers. Taken together, our data suggest that circulating MAIT cell deficiency might be characteristic of the development of MACs rather than non-MACs.

We hypothesized that reduced MAIT cells in peripheral blood might be caused due to their migration to the mucosal lesions in MACs from the circulation, rather than MAIT cell death or impaired MAIT cell proliferation. In the present study, MAIT cells were found to be more accumulated in colon cancer tissue than in the unaffected tissue, consistent with previous studies [14, 34]. Circulating MAIT cells have been reported to preferentially express CCR6, supporting their migration to the intestine [9]. Our data revealed that circulating MAIT cells exhibited higher levels of CCR6 and CXCR6, intermediate level of CXCR3, and lower level of CCR9 either in MAC patients or in healthy controls, which was consistent with a previous report [9]. Moreover, this is strongly supported by a previous study demonstrating that even colonic MAIT cells displayed high expression of CCR6 in patients with colorectal cancer [14]. Furthermore, we found that colon cancer tissues exhibited higher expressions of CCL20 and CXCL16, but similar expressions of CCL21 and CXCL10 as compared with unaffected tissue, which is in line with previous studies [35, 36]. Moreover, Transwell migration assay demonstrated that MAIT cells efficiently migrated via this chemokine-chemokine receptor axis. Interestingly, the expression of CCL20 and CXCL16 by tumor cells has been reported to be correlated with prognosis and infiltration of lymphocytes into cancer tissues [36, 37]. Collectively, these findings indicate that circulating CCR6 and CXCR6-expressing MAIT cells can be easily attracted into the cancer tissues releasing the corresponding chemokines, such as CCL20 and CXCL16, respectively, in MAC patients.

We observed a significant inverse correlation between circulating MAIT cell numbers and N staging or CEA, indicating that circulating MAIT cell deficiency could reflect the degree of cancer progression in MAC patients. A recent study analyzing archival tumor tissues by confocal microscopy demonstrated that increased tumor infiltration by MAIT cells was correlated with poor survival in colorectal cancer patients [38]. This finding is also supported by a previous study showing that profiling immune cells in and around tumors might help stage cancers and predict survival [39]. Taken together, all these studies suggest that measurement of MAIT cells, irrespective of their residency (circulation or mucosal tissues), could provide information on the prognosis of MAC patients. Therefore, prospective studies using a large-scale cohort are needed to evaluate the prognostic value of circulating and tumor-infiltrating MAIT cells.

MAIT cells have been known to play an antimicrobial role [10], but little is known about MAIT cell function in tumors, and there is only one recent study yet [14]. In that study, tumor-infiltrating MAIT cells had a much lower capacity for IFN-γ secretion compared to those in unaffected tissues. However, there is no functional study of circulating MAIT cells. Thus, we compared the production of cytokines such as IFN-γ, IL-17, and TNF-α, which were known to have anti-cancer activity [28, 29, 40], between colon cancer patients and healthy controls. Our study showed that the proportion of circulating MAIT cells producing these cytokines was comparable between MAC patients and healthy controls, suggesting that circulating MAIT cells appeared to have antagonistic potential against cancer cells. On the other hand, it could be interpreted differently based on the notion that immune defense against cancer might be weakened because MAC patients actually have decreased absolute numbers of functional MAIT cells due to reduced total numbers of circulating MAIT cells. Interestingly, the current study showed that the supernatant of activated MAIT cells enhanced NK cytotoxicity. Our previous study showed that LAK activity was also observed in NKT cells, another innate T cell subset, following αGalCer stimulation. This phenomenon was prevented predominantly by anti-IFN-γ blocking antibody [41]. Furthermore, activated iNKT cells induce antitumor responses primarily due to the subsequent activation of NK cells and production of IFN-γ [42]. These findings provide evidence that LAK activity of MAIT cells may be predominantly mediated via IFN-γ. Taken together, we demonstrated the indirect anti-cancer activity of MAIT cells via NK cells targeting K562 cells.

Until now, evidence for a direct role of MAIT cells in the control of tumor growth has not been presented. Notably, we demonstrated that activation of purified MAIT cells in peripheral blood induced direct cytotoxicity on the K562 cancer cell line without NK cells. To the best of our knowledge, this is the first evidence supporting the claim that circulating MAIT cells can directly kill cancer cells. In general, cytotoxicity occurs primarily via exocytosis of cytotoxic granules containing perforin and granzyme, which are key granule proteins required for the granzyme transport channel and apoptosis through caspase activation, respectively [43]. A recent study showed that circulating MAIT cells were licensed to kill their cognate target cells upon bacterial stimulation, which is mediated by degranulation of granzyme B and perforin [44]. Our data also revealed that activation of circulating MAIT cells induced the degranulation of granzyme B and/or perforin in an either MR1-dependent or MR1-independent manner, respectively. This finding was supported by a recent study which showed that colonic MAIT cells secrete granzyme B in response to stimulation with PMA and IM [14]. Taken together, these results indicate that direct cytotoxicity of MAIT cells against cancer cells may be mediated via degranulation of granzyme B and perforin. Therefore, we defined a new role of MAIT cells as having an anti-cancer effect characterized by both LAK activity and direct cytotoxicity.

We firstly show that circulating MAIT cells are reduced in MAC patients due to migration to mucosa cancer tissues. These reduced circulating cells, capable of producing anti-cancer cytokines, can be considered prognostic markers in MAC patients. Notably, activated MAIT cells not only have LAK activity on cancer cells but they also have direct cytotoxicity, which may be mediated via degranulation of cytotoxic granules. It is highlighted that MAIT cells may be the new killer cells against cancers in mucosal oncoimmunity.

## MATERIALS AND METHODS

### Subjects

The study cohort included 20 HCs, 15 patients with gastric cancer, 34 patients with colon cancer, 13 patients with lung cancer, 13 patients with breast cancer, 6 patients with hepatocellular carcinoma, and 18 patients with thyroid cancer before treatment. The clinical and laboratory characteristics of the patients are summarized in Table 1. The study protocol was approved by the Institutional Review Board of Chonnam National University Hospital (CNUH-2014-130), and written informed consent was obtained from all participants in accordance with the Declaration of Helsinki.

### Monoclonal antibodies (mAbs) and flow cytometry

The following mAbs and reagents were used in this study: Allophycocyanin (APC)-Alexa Fluor 750-conjugated anti-CD3, phycoerythrin (PE)-Cy5-conjugated anti-CD161, fluorescein isothiocyanate (FITC)-conjugated anti-TCR γδ, FITC-conjugated anti-IFN-γ, PE-conjugated anti-IL-17, PE-Cy7-conjugated anti-TNF-α, PE-conjugated anti-CCR6, PE-conjugated anti-CCR9, PE-conjugated anti-CXCR3, PE-conjugated anti-CXCR6, FITC-conjugated anti-perforin, FITC-conjugated anti-granzyme, FITC-conjugated anti-CD107a, FITC-conjugated mouse IgG isotype, PE-conjugated mouse IgG isotype and PE-Cy7-conjugated mouse IgG isotype control (all from Becton Dickinson, San Diego, CA) and APC-conjugated anti-TCR Vα7.2 (BioLegend, San Diego, CA). Cells were stained with combinations of appropriate mAb for 20 minutes at 4°C. Stained cells were analyzed on a Navios flow cytometer using Kaluza software (Beckman Coulter, Brea, CA).

### Isolation of peripheral blood mononuclear cells (PBMCs) and MAIT cells

Peripheral venous blood samples were collected in heparin-containing tubes, and PBMCs were isolated by density-gradient centrifugation using Ficoll-Paque Plus solution (Amersham Bioscience, Uppsala, Sweden). MAIT cells were identified phenotypically as CD3+TCRγδ-Vα7.2+CD161^high^ by flow cytometry as previously described [10, 24, 45]. Total lymphocyte numbers were measured by Coulter LH750 automatic hematology analyzer (Beckman Coulter, Miami, FL). Absolute numbers of MAIT cells were calculated by multiplying the MAIT cell percentages by the CD3+γδ- T cell percentages and the total lymphocyte numbers (per microliter) in peripheral blood. For sorting of MAIT cells, PBMCs were stained with PE-conjugated anti-CD3, FITC-conjugated anti-TCR γδ, APC-conjugated anti-TCR Vα7.2 and PE-Cy5-conjugated anti-CD161 and sorted to obtain CD3+TCRγδ-Vα7.2+CD161^high^ MAIT cells using a FACS Aria I sorter (BD Biosciences, Mountain View, CA). MAIT cells were isolated at purities of > 98%.

### Cell preparation from cancer tissues

We obtained cancer and adjacent unaffected tissues from patients who underwent colorectomy for colorectal cancer at the Department of Colorectal Surgery, Chonnam National University Hospital. Cancer and adjacent unaffected tissues were taken from the exophytic component immediately after surgical removal, avoiding grossly necrotic areas. Fresh specimens were resected under sterile conditions with two identical portions. One portion of specimen was fixed with neutral buffered formalin and embedded in paraffin for immunofluorescence staining. For flow cytometry, single-cell suspension of the other one was prepared by mechanical disruption on 40 μm cell strainers within 30 minutes after resection from patients [10, 46].

### Cell culture and intracellular cytokine staining

IFN-γ, IL-17A and TNF-α expression in MAIT cells was detected by intracellular cytokine flow cytometry as previously described [9, 10, 22, 41, 47]. Briefly, freshly isolated PBMCs (1 × 10^6^/well) were incubated in 1 mL complete media, consisting of RPMI 1640, 2 mM L-glutamine, 100 units/mL of penicillin, and 100 μg/mL of streptomycin, and supplemented with 10% fetal bovine serum (FBS; Gibco BRL, Grand Island, NY) for 4 hours in the presence of phorbol myristate acetate (PMA) (100 ng/mL; Sigma, St Louis, MO) and ionomycin (IM) (1 μM; Sigma). For intracellular cytokine staining, 10 μL of brefeldin A (GolgiPlug; BD Biosciences, San Diego, CA) was added, and the final concentrations of brefeldin A were 10 μg/mL. After incubation for an additional 4 hours, cells were stained with APC-Alexa Fluor 750-conjugated anti-CD3, PE-Cy5-conjugated anti-CD161 and APC-conjugated anti-TCR Vα7.2 mAb for 20 minutes at 4°C, fixed in 4% paraformaldehyde for 15 minutes at room temperature, and permeabilized with Perm/Wash solution (BD Biosciences) for 10 minutes. Cells were then stained with FITC-conjugated anti-IFN-γ, PE-conjugated anti-IL-17 or PE-Cy7-conjugated anti- TNF-α mAbs for 30 minutes at 4°C and analyzed by flow cytometry.

### Immunofluorescence staining

MAIT cells were detected by Immunofluorescence staining in tissues as previously described [10, 48]. Cancer tissues were fixed with neutral buffered formalin and embedded in paraffin. Deparaffinized sections were hydrated and then incubated with rat anti-human CD8 mAb at 1 : 100 (Bio-Rad, Hercules, CA), mouse anti-human TCR Vα7.2 mAb at 1 : 100 (Biolegend), and goat anti-human IL-18Rα at 1 : 100 (R&D systems, Inc., Minneapolis, MN) overnight at 4°C, washed with phosphate buffered saline (PBS), and treated with Alexa Fluor 568 goat anti-rat IgG, Alexa Fluor 488 goat anti-mouse IgG and Alexa Fluor 350 donkey anti-goat IgG (all from Life Technologies, Carlsbad, CA). Sections were mounted using Vector-shield mountant (Vector) and analyzed by LSM 510 confocal laser microscopy (Zeiss, Jena, Germany).

### RT-PCR and real-time PCR

Total RNA was isolated with TriZol reagent (Invitrogen, Carlsbad, CA). First-strand cDNA was transcribed from 1 μg of RNA using Superscript RT (Invitrogen), according to the manufacturer's instructions. Reverse-transcribed cDNA samples were then added to a PCR mixture consisting of 10× PCR buffer, 0.2 mM dNTPs, 0.5 units of *Taq* DNA polymerase (Takara, Tokyo, Japan), and 10 pmoles of primers for each gene. The sequences of the primers used were as follows: for β-actin, 5′-CTCCTTAATGTCACGCACGAT-3′ (sense) and 5′-GTGGGGCGCCCCAGGCACCA-3′ (antisense); for CCL20, 5′-CTGGCTGCTTTGATGTCAGT-3′ (sense) and 5′- CGTGTGAAGCCCACAATAAA-3′ (antisense); for CCL21, 5′-GCCTTGCCACACTCTTTC TC-3′ (sense) and 5′- CAAGGAAGAGGTGGGGTGTA-3′ (antisense); for CXCL10, 5′-GGAACCTCCAGTCTCAGCACC-3′ (sense) and 5′-CAGCCTCTGTGTGGTCCAATCC-3′ (antisense); for CXCL16, 5′-GACATGCTTACTCGGGGATTG-3′ (sense) and 5′-CAGTGATCCTACTGGGAGGGT-3′ (antisense). Amplifications were conducted over 27 cycles at 94°C for 30 seconds (denaturation), and 55°C for 30 seconds (annealing), 72°C for 30 seconds (extension). This was followed by an additional extension step at 72°C for 10 minutes in a PCR cycler (Bio-Rad). PCR products were subjected to electrophoresis and visualized by ethidium bromide staining. Real-time PCR was performed using QuantiTect SYBR Green PCR kits (Qiagen, Valencia, CA) in triplicate in a Rotor-Gene 3000 (Corbett Research, Mortlake, NSW, Australia), using the following conditions; 15 minutes at 95°C, followed by 40 amplification cycles at 95°C for 30 seconds, 58°C for 30 seconds, and 72°C for 30 seconds. All quantifications were normalized versus endogenous β-actin. The relative quantitation value of each target gene after normalizing to β-actin as compared with the calibrator for that target was calculated using 2^−(Ct-Cc)^(Ct and Cc are the mean threshold cycle differences after normalizing to β-actin).

### Transwell migration assay

Migration of T cells and MAIT cells through Transwell membrane was performed using modified methods as previously described [49, 50]. Briefly, T cells were purified from PBMCs by negative selection using Pan T Cell Isolation Kit (Miltenyi Biotec, Bergisch Gladbach, Germany), according to the manufacturer's instructions. Sorted T cells (5 × 10^5^ cells/well) were added to upper chambers of Transwell plates (5-μm pore size; Costar, Cambridge, MA). Recombinant human (rh) CCL20 (200 ng/ml; PeproTech, London, UA), rhCXCL16 (200 ng/ml; PeproTech), mixture of rhCCL20 and rhCXCL16 (200 ng/ml), rhCCL21 (200 ng/ml; PeproTech) and PBS were added to lower chambers containing serum free RPMI1640 media. Plates were incubated for 90 minutes at 37°C. Migrated cells were stained with APC-Alexa Fluor 750-conjugated anti-CD3, FITC-conjugated anti-TCR γδ, APC-conjugated anti-TCR Vα7.2 and PE-Cy5-conjugated anti-CD161 mAbs and then counted by Neubauer chamber and flow cytometer. Migration index was calculated by the number of cells that migrated in the presence of each chemokine divided by the number of cells that migrated in the presence of PBS. The migration index of MAIT cells was normalized to the migration index of T cells.

### Cytotoxicity

MAIT cells and K562 cells (CCL-243; ATCC) were used as effector and target cells, respectively. MAIT cytotoxicities were evaluated by flow cytometry at an effector-to-target (E:T) cell ratio of 40:1 as previously described [51, 52]. Briefly, purified MAIT cells (1 × 10^6^/well) were cocultured with K562 cells for 4 hours following stimulation with or without PMA (100 ng/ml; Sigma, St. Louis, MO) and ionomycin (IM; 1 μM; Sigma) for 12 hours. Mixed effector and target cells were then stained with FITC-conjugated anti-CD45 mAb at 4°C for 20 minutes, washed once in phosphate buffered saline (PBS), resuspended in 0.5ml of PBS containing 20 μl of 1 μg/ml propidium iodide (Becton Dickinson), and incubated at room temperature for 15 minutes. Percentages of dead K562 cells were determined by flow cytometry.

To determine LAK activity of MAIT cells, purified MAIT cells were incubated for 12 hours in the presence or absence of PMA (100 ng/ml) and IM (1 μM). Culture supernatants were harvested from the purified MAIT cells and then added to tubes including PBMCs and K562 cells. LAK activities of PBMCs were evaluated by flow cytometry and determined at an E:T cell ratio of 20:1.

### Granzyme and perforin expression

Granzyme and perforin expression in MAIT cells was detected by intracellular cytokine flow cytometry as previously described [14, 44, 53]. Briefly, freshly isolated PBMCs (1 × 10^6^/well) were incubated for 12 hours in the presence of PMA (100 ng/ml) and IM (1 μM) or for 24 hours with E. coli-infected THP-1 cells (1 × 10^6^/well; American Type Culture Collection). For intracellular staining, 10 μL of brefeldin A (GolgiPlug; BD Biosciences, San Diego, CA) was added, and the final concentrations of brefeldin A were 10 μg/mL. After incubation for an additional 4 hours, cells were stained with PE-conjugated anti-CD3, PE-Cy5-conjugated anti-CD161 and APC-conjugated anti-TCR Vα7.2 mAb for 20 minutes at 4°C, fixed in 4% paraformaldehyde for 15 minutes at room temperature, and permeabilized with Perm/Wash solution (BD Biosciences) for 10 minutes. Cells were then stained with FITC-conjugated anti-Granzyme and FITC-conjugated anti-perforin mAbs for 30 minutes at 4°C and analyzed by flow cytometry.

### CD107a degranulation assay

The degranulation of MAIT cells in response to cancer cell lines was determined by flow cytometry as previously described [54–56]. Briefly, freshly isolated PBMCs (1 × 10^6^/well) were incubated for 4 hours in the presence of PMA (100 ng/ml) and IM (1 μM) or for 24 hours with *E. coli*-infected THP-1 cells (1 × 10^6^/well). The cells were stained with FITC-conjugated anti-CD107a or isotype control mAb and then incubated with or without K562 at an E:T ratio of 20:1. After 1 hour, monensin (GolgiStop; BD Biosciences, San Diego, CA) and brefeldin A were added, and the cells were incubated for an additional 4 hours at 37°C in 5% CO2. After the culture, the cells were stained with PE-conjugated anti-CD3, APC-conjugated anti-TCR Vα7.2 and PE-Cy5-conjugated anti-CD161 mAb for 20 minutes at 4°C, fixed in 4% paraformaldehyde for 15 minutes at room temperature and then analyzed by flow cytometry.

### Statistical analysis

All comparisons of percentages, absolute numbers and cytokine levels of MAIT cells in peripheral blood were performed by analysis of covariance after adjusting for age and sex using Bonferroni correction for multiple comparisons. Spearman's correlation coefficients were used to examine the relationships between MAIT cell numbers and clinical or laboratory parameters. Wilcoxon matched-pairs signed rank test was used for the comparison of MAIT cell levels between peripheral blood, cancer tissue, and adjacent unaffected tissue. Comparisons of chemokines, migration indices, granzyme, perforin and CD107a levels were examined using paired t test. *P* values less than 0.05 were considered statistically significant. Statistical analysis was performed using SPSS version 17.0 software.

## SUPPLEMENTARY MATERIAL TABLE


