# Supplementary material for: Clinical relevance of circulating mucosal-associated invariant T cell levels and their anti-cancer activity in patients with mucosal-associated cancer
Source: Oncotarget. 2016 Aug 10;7(46):76274–90. doi: 10.18632/oncotarget.11187 (PMC5342813; doi:10.18632/oncotarget.11187)
Supplement: Supplementary file 1 [file oncotarget-07-76274-s001.pdf]

Clinical relevance of circulating mucosal-associated invariant T cell levels and their anti-cancer activity in patients with mucosal-associated cancer

Supplementary Material

Supplementary Table 1. Spearman's correlation coefficients for the percentage of tumor-infiltrating MAIT cells with respect to clinical and laboratory findings in 17 colon cancer patients.

Variable	Correlation coefficient (γ_s)	*P* value
Age	-0.242	0.385
T staging	-0.149	0.596
N staging	-0.405	0.135
Tumor size	-0.589	0.021
Leukocyte count (cells/μL)	-0.082	0.771
Lymphocyte count (cells/μL)	0.234	0.401
Monocyte count (cells/μL)	-0.538	0.038
Neutrophil count (cells/μL)	-0.043	0.879
Hemoglobin (g/dL)	0.429	0.111
Platelet count (10^3 cells/μL)	0.080	0.776
AST (U/L)	0.075	0.790
ALT (U/L)	0.098	0.728
BUN (mg/dL)	-0.434	0.106
Creatinine (mg/dL)	-0.391	0.150
Total protein (g/dL)	0.364	0.182
Albumin (g/dL)	0.547	0.035
CRP (mg/dL)	-0.482	0.069
CEA (ng/mL)	-0.174	0.553

Abbreviations: AST, Aspartate transaminase; ALT, Alanine transaminase; BUN, Blood urea nitrogen; CRP, C-reactive protein; CEA, Carcinoembryonic antigen; MAC, Mucosal-associated cancer.
